# Estimulação Transcraniana por Corrente Contínua no Tratamento da Hipertensão: Uma Hipótese Plausível?

**DOI:** 10.36660/abc.20230100

**Published:** 2023-09-19

**Authors:** Fernando Zanela da Silva Arêas, Elizangela Kuster, Lenon Corrêa de Souza, Wagner Jorge Ribeiro Domingues, João Siqueira, Luíz Henrique Aquino Serudo, Guilherme Peixoto Arêas

**Affiliations:** 1 Universidade Federal do Espírito Santo Vitória ES Brasil Universidade Federal do Espírito Santo – Neurociências – Ciências Fisiológicas, Vitória, ES – Brasil; 2 Universidade Federal do Amazonas Instituto de Educação em Ciências Sociais e Zootecnia Manaus AM Brasil Universidade Federal do Amazonas – Instituto de Educação em Ciências Sociais e Zootecnia, Manaus, AM – Brasil; 3 Universidade Federal do Amazonas Departamento de Fisiologia Manaus AM Brasil Universidade Federal do Amazonas – Departamento de Fisiologia, Manaus, AM – Brasil; 4 Universidade Federal do Amazonas Manaus AM Brasil Universidade Federal do Amazonas, Manaus, AM – Brasil

**Keywords:** Hipertensão, Sistema Nervoso Autônomo, Estimulação Elétrica Nervosa Transcutânea

A hipertensão arterial (HAS) é a doença crônica mais prevalente em todo o mundo,^1^ sendo um dos mais importantes fatores de risco cardiovascular.^2^ Dentre as diversas variáveis que controlam a pressão arterial (PA), aquelas que interagem diretamente com os mecanismos neurais envolvem múltiplas áreas do sistema nervoso central (SNC).^3^

A relação entre a atividade cerebral e a hipertensão foi demonstrada por estudos neurofisiológicos em animais, mostrando que a estimulação elétrica ou química direta de diferentes estruturas do cérebro e do córtex cerebral pode modificar significativamente a PA e a frequência cardíaca (FC).^4-6^

Além disso, os sistemas simpático e renal parecem contribuir para as alterações vasculares que ocorrem inicialmente na hipertensão, como aumento da resistência vascular basal mantida pelo remodelamento dos vasos e redução do número de vasos.^7^ Ademais, a redução da PA periférica normalmente falha em reverter as alterações na função, estrutura e organizações cerebrais associadas à doença, sugerindo que as influências no cérebro não são secundárias à elevação da PA.^7^ As causas iniciais exatas da hipertensão permanecem obscuras, e a origem multifatorial é a principal hipótese. Assim, não podemos dizer que o cérebro inicia a hipertensão, mas é claro que a hipertensão prejudica o funcionamento do cérebro. Portanto, mais estudos são necessários para confirmar se a hipertensão se origina principalmente como uma doença cerebral ou uma doença da vasculatura periférica.

A revisão de Jennings e Zanstra (2009)^7^ demonstra que a hipertensão está associada ao suporte do fluxo sanguíneo cerebral (FSC) alterado para o processamento cognitivo. Em suma, a hipertensão parece induzir uma leve redução do FSC, que pode ser mais acentuada nas regiões frontal e subcortical.

Dada a relação entre PA e atividade cortical, as técnicas de estimulação cerebral parecem ser uma ferramenta potencialmente útil a ser explorada. A estimulação transcraniana por corrente contínua (ETCC) é um tipo de técnica de estimulação cerebral não invasiva (ECNI) que se refere à aplicação de uma corrente contínua através de um par de eletrodos de superfície (ânodo e cátodo).^3^

Estudos em animais e humanos forneceram informações sobre os mecanismos subjacentes aos efeitos da ETCC na neuroplasticidade e mostraram que a ETCC pode induzir alterações específicas na atividade neuropsicológica, psicofisiológica e motora, dependendo da área cerebral alvo.^8^ No entanto, uma revisão sistemática mostrou que a heterogeneidade dos estudos impossibilitou a extração de evidências conclusivas sobre os efeitos da ECNI nos sistemas cardiovascular e autonômico.^9^ Isso ocorre principalmente porque a maioria dos estudos existentes foi desenvolvida para entender a segurança da ECNI usando parâmetros cardiovasculares e não para estudar a conexão cérebro-coração.^3^

As técnicas de ECNI que têm sido mais intensamente estudadas em relação ao sistema nervoso autônomo e cardiovascular são a estimulação magnética transcraniana repetitiva (rTMS) e a ETCC. A ETCC modula a atividade espontânea da rede neural aplicando correntes elétricas fracas em diferentes áreas corticais.^3^ No nível neuronal, o principal mecanismo de ação é a indução de alterações dependentes da polaridade na excitabilidade cortical.^10^

Embora o posicionamento dos eletrodos ainda não esteja totalmente resolvido devido a influências em áreas adjacentes após o estímulo, o seguinte posicionamento é o International EEG 10-20 System. É onde um eletrodo ânodo e um eletrodo cátodo são colocados na pele visando a modulação de uma área focal pré-determinada do córtex.^8^

A principal teoria é que a ECNI poderia modular o fluxo simpático do tronco cerebral a partir da atividade neural de áreas corticais autônomas, como o córtex sensório-motor, o córtex pré-frontal medial (CPFM) e o córtex insular ou regiões hipotalâmicas por meio de projeções do córtex pré-frontal. O CPFM pode ser o alvo mais adequado para a aplicação da ECNI, pois possui várias projeções para as principais localizações do tronco encefálico envolvidas na função autonômica e também porque a estimulação elétrica e química direta do CPFM está associada a respostas simpáticas inibitórias. Nesta estrutura, pode-se supor que a aplicação de ETCC anódica de excitabilidade aumentada ativaria essa área-alvo, que modularia a PA por inibição da atividade simpática.^11^

Portanto, a justificativa para nossa hipótese é que o uso de ETCC pode ser capaz de modular a PA sistêmica por meio de estímulos no córtex cerebral.^12^ Dessa forma, procuramos elucidar, de forma experimental, o melhor entendimento desse mecanismo de controle e investigar uma possível nova alternativa para o controle da PA e tratamento da hipertensão.

Um primeiro estudo de Rodrigues et al.^13^ composto por 13 participantes com HAS, avaliou a efetividade de uma intervenção aguda de ETCC. Confirmou-se a hipótese de que uma única sessão de ETCC pode reduzir a atividade simpática e melhorar a modulação autonômica cardiovascular levando a uma redução da PA por 24 horas.

Outro estudo que avaliou a eficácia a curto prazo da ETCC em 20 participantes foi realizado por Silva-Filho et al.^14^ onde os autores observaram redução da PA sistólica e diastólica durante o sono e três horas após a intervenção. No entanto, nada sugeriu mudanças significativas nos valores de variabilidade.

Um terceiro estudo de Rodrigues et al.^15^ envolveu 13 participantes com HAS resistente. Com um intervalo de pouco menos de 1 mês, avaliou uma intervenção aguda e uma intervenção de curto prazo. Durante a intervenção aguda, observou-se que a estimulação leva à redução dos valores hemodinâmicos, como débito cardíaco, resistência vascular periférica e PA sistólica, diastólica e média. Esses resultados foram mantidos 24 horas após a estimulação. Em contraste, a intervenção de curto prazo não forneceu evidências suficientemente fortes de redução dos valores hemodinâmicos ligados à PA, mas a modulação autonômica teve respostas positivas após as sessões.

Todos os autores citados encontraram dificuldades não relatadas na realização dos estudos. No entanto, um ponto comum entre os artigos é que os resultados às vezes são inconclusivos, provavelmente devido ao pequeno tamanho da amostra, curto tempo de intervenção e não padronização do protocolo de aplicação.

O córtex cerebral é uma estrutura fundamental para o controle cardiovascular, pois o sistema nervoso está relacionado ao controle da PA e à patogênese de diversas formas de hipertensão.^11^ Também é importante destacar o papel do hipotálamo, especificamente o núcleo paraventricular, na mediação das respostas endócrinas e autonômicas que mantêm a pressão sanguínea e a homeostase do fluido corporal por meio de fenótipos neuronais distintos que controlam os eixos endócrinos e o fluxo simpático. Embora existam alguns tratamentos para hipertensão, uma porcentagem substancial de pacientes sofre de hipertensão “resistente a medicamentos”, uma condição associada ao aumento da ativação cerebral dos receptores de angiotensina, aumento da atividade do sistema nervoso simpático e níveis elevados de vasopressina.

*********************

Evidências mostram que os neurônios aumentam a expressão do receptor de angiotensina Tipo 1a localizado dentro da lâmina terminal por meio da regulação endócrina e respostas comportamentais que estão envolvidas na manutenção da homeostase cardiovascular, revelando assim conexões excitatórias funcionais entre os neurônios sensíveis à angiotensina nos terminais da lâmina e os neurônios da vasopressina no núcleo paraventricular do hipotálamo, indicando ainda que a ativação desse circuito aumenta a PA. Assim, esses neurônios também podem ser um alvo promissor para a terapia anti-hipertensiva.^16^

Por fim, resultados de estudos em animais obtêm ampla margem de melhora na aplicação das técnicas de ECNI. Assim, a aplicação dessas técnicas em humanos para pesquisas e tratamentos clínicos poderiam ser baseados nos achados que faremos para otimizar e refinar o protocolo de estimulação.^14^
