# Supplementary material for: Estimulação Transcraniana por Corrente Contínua no Tratamento da Hipertensão: Uma Hipótese Plausível?
Source: Arq Bras Cardiol. 2023 Sep 19;120(9):e20230100. [Article in Portuguese] doi: 10.36660/abc.20230100 (PMC10519356; doi:10.36660/abc.20230100)
Supplement: Translation

# Translation: Transcranial Direct Current Stimulation in the Management of Hypertension: A Plausible Hypothesis?

**Authors:** Fernando Zanela da Silva Arêas, Elizangela Kuster, Lenon Corrêa de Souza, Wagner Jorge Ribeiro Domingues, João Siqueira, Luíz Henrique Aquino Serudo, Guilherme Peixoto Arêas

**Affiliations:** 1 Brasil Universidade Federal do Espírito Santo – Neurociências – Ciências Fisiológicas, Vitória, ES – Brasil; 2 Brasil Universidade Federal do Amazonas – Instituto de Educação em Ciências Sociais e Zootecnia,Manaus, AM – Brasil; 3 Brasil Universidade Federal do Amazonas – Departamento de Fisiologia, Manaus, AM – Brasil; 4 Brasil Universidade Federal do Amazonas, Manaus, AM – Brasil

**Keywords:** Hypertension, Autonomic Nervous System, Transcutaneous Electric Nerve Stimulation

Hypertension (HTN) is the most prevalent chronic disease in the entire world,^1^ being one of the most significant cardiovascular risks factors.^2^ Among several variables controlling blood pressure (BP), those directly interacting with neural mechanisms involve multiple areas of the central nervous system.^3^

The relationship between brain activity and HTN has been demonstrated by neurophysiological studies on animals showing that direct electrical or chemical stimulation of different structures of the brainstorm and cerebral cortex can significantly modify BP and heart rate.^4-6^

In addition, the sympathetic and renal systems seem to contribute to the vascular changes that occur initially in HTN, such as increased basal vascular resistance maintained by remodeling the vessels and reducing the number of vessels.^7^ Furthermore, reducing peripheral BP normally fails to reverse changes in brain function, structure, and organizations associated with the disease, suggesting that influences on the brain are not secondary to elevation of BP.^7^ The exact initial causes of HTN remain unclear, and a multifactorial origin is the main hypothesis. Thus, we cannot say that the brain initiates HTN, but it is clear that HTN harms the brain’s functioning. Therefore, more studies are needed to confirm whether HTN originates mainly as a brain disease or a disease of the peripheral vasculature.

The review by Jennings and Zanstra (2009)^7^ demonstrates that HTN is associated with the support of altered cerebral blood flow (CBF) for cognitive processing. In short, HTN seems to induce a slight reduction in CBF, which may be more accentuated in the frontal and subcortical regions.

Given the relationship between BP and cortical activity, brain stimulation techniques appear to be a potentially useful tool to be explored. Transcranial direct current stimulation (tDCS) is a type of non-invasive brain stimulation technique (NIBS) that refers to the application of a direct current through a pair of surface electrodes (anode and cathode).^3^

Studies on animals and humans have provided information on the mechanisms underlying the effects of tDCS on neuroplasticity and have shown that tDCS can induce specific changes in neuropsychological, psychophysiological, and motor activity depending on the targeted brain area.^8^ However, a systematic review showed that the heterogeneity of the studies made it impossible to extract conclusive evidence about the effects of NIBS on the cardiovascular and autonomic systems.^9^ This is mainly because most existing studies were developed to understand the safety of NIBS using cardiovascular parameters and not to study brain-heart connection.^3^

The NIBS techniques that have been most intensively studied regarding the cardiovascular and autonomic nervous system are repetitive transcranial magnetic stimulation and tDCS. The tDCS modulates the neural network’s spontaneous activity by applying weak electrical currents in different cortical areas^3^. At the neuronal level, the main mechanism of action is the induction of polarity-dependent changes in cortical excitability.^10^

Although the positioning of the electrodes is not yet completely resolved due to influences on adjacent areas after the stimulus, the following positioning is the International EEG 10-20 System. This is where an anode electrode and a cathode electrode are placed on the skin targeting the modulation of a predetermined focal area of the cortex.^8^

The main theory is that NIBS could modulate sympathetic brain stem flow from the neural activity of autonomic cortical areas such as the sensorimotor cortex, the medial prefrontal cortex (MPFC), and the insular cortex, or hypothalamic regions via projections of the prefrontal cortex. MPFC may be the most suitable target for the application of NIBS since it has several projections for the main brain stem locations involved in autonomic function, and also because direct electrical and chemical stimulation of MPFC are associated with inhibitory sympathetic responses. In this structure, it can be assumed that the application of anodic tDCS of increased excitability would activate this target area, which would modulate BP by inhibiting sympathetic activity.^11^

Therefore, the rationale for our hypothesis is that the use of tDCS may be able to modulate systemic BP through stimuli in the cerebral cortex.^12^ In this way, we attempt to elucidate, in an experimental approach, the best understanding of this control mechanism and investigate a possible new alternative for controlling BP and treating HTN.

A first study by Rodrigues et al.^13^ composed of 13 participants with arterial HTN, evaluated the effectiveness of an acute intervention of tDCS. It confirmed the hypothesis that a single session of tDCS can reduce sympathetic activity and improve cardiovascular autonomic modulation leading to a reduction in BP for 24 hours.

Another study that evaluated the short-term effectiveness of tDCS in 20 participants was performed by Silva-Filho et al.^14^ where the authors observed a reduction in systolic and diastolic BP during sleep and three hours after the intervention. However, nothing suggested significant changes in the variability values (HRV).

A third study by Rodrigues et al.^15^ involved 13 participants with resistant arterial HTN. At an interval of just under 1 month, it evaluated an acute intervention and a short-term intervention. During the acute intervention, it was observed that stimulation leads to a reduction in hemodynamic values, such as cardiac output, peripheral vascular resistance, and systolic, diastolic, and mean BP. These results were maintained 24 hours after stimulation. In contrast, the short-term intervention did not provide strong enough evidence of a reduction in hemodynamic values linked to BP, but the autonomic modulation had positive responses after the sessions.

All the cited authors found unreported difficulties in carrying out the studies. However, a common point among the articles is that the results are sometimes inconclusive, probably due to the low sample size, short intervention time, and non-standardization of the application protocol.

The cerebral cortex is a fundamental structure for cardiovascular control because the nervous system is related to controlling BP and the pathogenesis of various forms of HTN.^11^ It is also important to highlight the role of the hypothalamus, specifically the paraventricular nucleus (PVN), in mediating the endocrine and autonomic responses that maintain BP and body fluid homeostasis through distinct neuronal phenotypes that control endocrine axes and sympathetic outflow. Although there are some treatments for HTN, a substantial percentage of patients suffer from “drug resistant” HTN, a condition associated with increased brain activation of angiotensin receptors increased sympathetic nervous system activity and elevated vasopressin levels.

Evidence has shown that neurons increase the expression of the Type 1a angiotensin receptor located within the terminal lamina through endocrine regulation and behavioral responses that are involved in maintaining cardiovascular homeostasis, Thereby revealing functional excitatory connections between angiotensin-sensitive neurons at the lamina terminals and vasopressin neurons in the paraventricular nucleus of the hypothalamus, and further indicating that the activation of this circuit increases BP. Thus, these neurons may also be a promising target for antihypertensive therapy.^16^

Finally, results of studies in animals obtain a wide margin of improvement in the application of NIBS techniques. Thus, the application of these techniques in humans for research and clinical treatments could be based on the findings that we will make to optimize and refine the stimulation protocol.^14^
